# An Experimental Study of the Mechanical Properties of Partially Rehabilitated Cable Tunnels

**DOI:** 10.3390/ma15144830

**Published:** 2022-07-11

**Authors:** Zihao Zhu, Baosong Ma, Zheng Zeng, Chenkun Gong, Zhe Mei, Jinqiu Hu, Peng Zhang

**Affiliations:** 1 Faculty of Engineering, China University of Geoscience-Wuhan, Wuhan 430074, China; zhuzihaocug@163.com (Z.Z.); z.zheng@cug.edu.cn (Z.Z.); 18086486825@cug.edu.cn (C.G.); haozhu@cug.edu.cn (Z.M.); cughujinqiu@163.com (J.H.); 2 School of Civil Engineering, Sun Yat-sen University, Guangzhou 510275, China; mabaos@mail.sysu.edu.cn

**Keywords:** cable tunnel, reinforced concrete pipe, partial rehabilitation, three-edge bearing test, cement mortar, epoxy resin

## Abstract

For buried municipal tunnels—such as cable tunnels and utility tunnels with structural defects—due to the sheltering of the internal pipelines, shelves, and other auxiliary facilities, traditional trenchless rehabilitating methods are not applicable since an intact ring is needed for spraying and lining. In these tunnels, only the exposed area at the crown of the ring can be partly rehabilitated. In this paper, three-edge bearing tests (TEBTs) for partially rehabilitated reinforced concrete (RC) pipe sections are carried out to simulate the case of a municipal tunnel and the effects of different repair materials (cement mortar and epoxy resin) and different dimensional parameters of the liner (lining thickness, lining range) on the partial rehabilitation effect of defective RC pipes are studied. The deforming compatibility of the liner–pipe interface is discussed, and the flexural rigidity of the partially rehabilitated section is calculated. The results show that the load-carrying capacities of partial rehabilitated RC pipes are effectively improved.

## 1. Introduction

**Figure 1:**
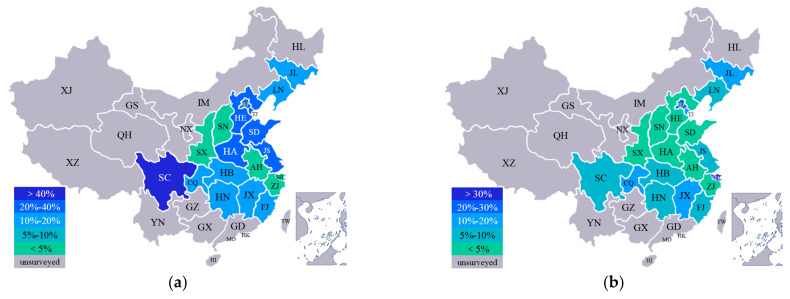
The ratio of (**a**) cracked and (**b**) corroded cable tunnels in different regions of China.

In recent years, the annual average growth of underground cable in China has reached 15%, and the milage of high-voltage cable tunnels has exceeded 15,000 km [1,2]. As a result, more and more structural problems have emerged that greatly threaten their safe maintenance and operation. The most common structural problems in cable tunnels include cracking and corroding [3,4,5], which has spread all over the major cities of central and eastern China, as shown in Figure 1. In some areas (i.e., Shanghai and Sichuan), the ratio of cable tunnels with cracks and corroded walls has reached 30–40%.

In most defective tunnels, small-scale cracking and corroding govern the structural distress; for these tunnels, the method of partial lining is widely applied, which is to mend the defective areas with cement mortar or polymeric materials to resist further defects and to restore the original wall thickness. This mending method is actually different from the concept of trenchless local rehabilitation methods, the latter of which includes the PVC segment method, local CIPP (cured-in-place-pipe) method, and point grouting method [6,7]. These methods mean to repair a localized whole ring in a continuous pipe section, whereas the partial rehabilitation method mentioned in this paper is to rehabilitate the arch of an intact ring. Consequently, this method is usually considered as a functional repairment rather than a structural rehabilitation, which also leads to the research blank on this method.

At present, most research is concerned about lining materials, including inorganic materials (cement mortar) and organic polymer materials (polyurethane, acrylate, epoxy resin, etc.). For the sake of environmental protection, the research on plain cement mortar mainly focuses on how to use industrial waste such as fly ash to replace the aggregate in cement mortar and thus reduce carbon emissions. Bae [8] studied the characteristics of mortars with blast furnace slag powder and mixed fine aggregates containing ferronickel-slag aggregate. Cobos [9] studied the influence of crystalline admixtures at early age performance of cement-based mortar by electrical resistance monitoring, and Fayed [10] proposed an innovative method for sustainable utilization of blast-furnace slag in the cleaner production of one-part hybrid cement mortar. On the other hand, many researchers have studied the high-performance cement mortar materials: Li [11] studied the mechanical properties of aramid/carbon hybrid fiber-reinforced concrete; Haroon [12] studied the performance of reinforced concrete beams strengthened with carbon fiber reinforced polymer strips; and Shi [13] studied the effect of desert sand on the pore structure of fiber reinforced mortar. As for polymer materials, most research has focused on the polymer materials modified with fibers and inorganic materials: Li [14] studied the anisotropic mechanical response and strain localization of a metallic glassy-fiber-reinforced polyethylene terephthalate fabric; Lionetto [15] studied carbon fiber reinforced polymers; Park [16] studied a microwave composite forming process based on a sic mold for manufacturing fiber metal laminate; and Lou [17] investigated the fatigue performance of an asphalt mixture reinforced by basalt fiber.

From the aspect of tunnel lining and reinforcement, Nie [18] analyzed the piezo resistivity for an inner-adhesive-type carbon fiber reinforced plastic tunnel reinforcement; Zhou [19] studied the dynamic response of the lining structure in a long tunnel subjected to a non-uniform seismic load; Sun [20] studied the spraying construction method of a non-water reacting polymer layer in the tunnel; Zheng [21] discussed the use of yielding elements in shotcrete linings in tunnel squeezing deformation control; Wang [22] studied the response characteristics of cross tunnel lining under a dynamic train load; and Zhang [23] studied the sustainability performance of reinforced concrete structures in tunnel lining induced by a long-term coastal environment.

**Figure 2:**
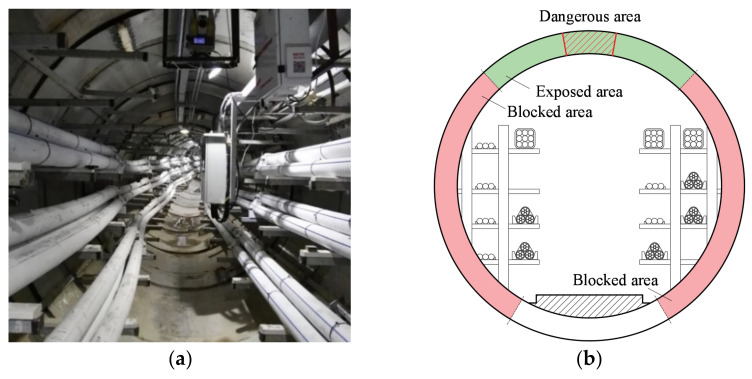
(**a**) The typical layout in a buried cable tunnel (Fujian, China) and (**b**) the sketch map of the cross section.

However, when it comes to municipal tunnels, due to the sheltering of the pipelines, shelves, and other auxiliary facilities (as shown in Figure 2a), the lateral wall of the pipe section is blocked and unable to be lined or sprayed with traditional trenchless rehabilitating methods [24], these areas are shown as the red area in Figure 2b. In most cases, the pipelines are not allowed to be removed or disconnected, as well as the shelves and the instruments. Therefore, the exposed area at the crown of the pipe becomes the only area for rehabilitation, which is shown as the green area in Figure 2b, and the partial lining method is bound to become a major rehabilitation method in these structures; thus, great importance is attached to the studying of partial lining materials and dimensional parameters for a better effect.

It can be learnt from previous studies [25,26,27] that for a pipe ring structure, the dangerous sections are located at the crown, invert, and springlines of the pipe under surface load, where the cracks appear and propagate first [28,29]. As shown in Figure 2b, the exposed area contains the dangerous section at the crown of the pipe, and it can be rehabilitated with the partial lining method, which suggests a potential structural enhancement by rehabilitating with this method [30,31]. To verify this hypothesis, several pre-cracked reinforced concrete pipe (RCP) models were cast as simulated tunnel sections, and different materials were applied for partial lining at the cracked area of the crown. After the rehabilitation, three-edge bearing tests (TEBTs) were carried out to test the load-carrying capacity of the specimens, with the results of which the effect of the partial lining method was evaluated.

## 2. Experimental Study

### 2.1. Test Regime

The designing and the coding of the six TEBTs are shown in Table 1, which includes four defective RCP specimens (denoted as T1–T4) and two control groups (T0-1 and T0-2). In test T1–T4, different materials and the shapes of the liners were studied. For the different shapes of the liners, two conditions were included (condition 1: 45° lining angle and 10 mm lining thickness; condition 2: 90° lining angle and 20 mm lining thickness). As for the materials, cement mortar (CM) and epoxy resin (ER) were chosen for the partial lining. Besides, an undamaged RCP (T0-1) and a damaged RCP without rehabilitation (T0-2) were also included as control groups.

### 2.2. Specimen and Instruments

#### 2.2.1. Pipe Specimens

**Figure 3:**
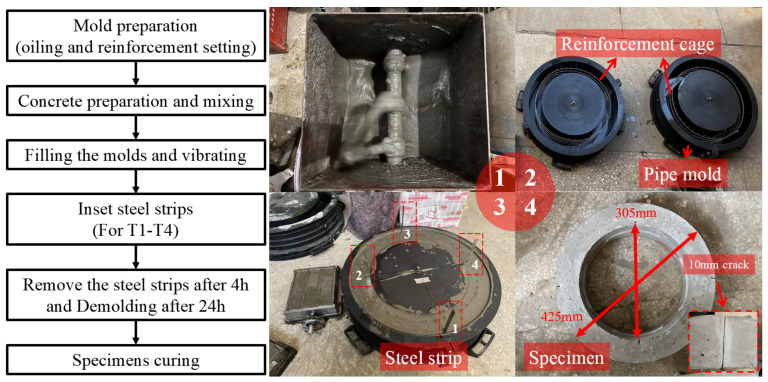
Procedures of RCP casting. (1. Mixing concrete; 2. Oiling mold and setting reinforcement; 3. Filling mold; 4. Demolding).

To ensure the stability of the material’s properties, all pipe specimens were made in a laboratory with the same batch of concrete and steel reinforcement, and all specimens were cast and cured at the same time and in the same environment; a flowchart and the procedures for the preparation of pipe specimens are shown in Figure 3. Firstly, the concrete was completely mixed and filled into the mold with a 305 mm inner diameter and a 425 mm outer diameter, the molds were oiled with mineral oil for the convenience of demolding; then, after the vibrating, four steel strips with a length of 150 mm, a width of 10 mm, and a thickness of 0.4 mm were inserted into the concrete at the internal edge of the crown and the invert and the external edges of the springlines of the RCPs, as shown in Figure 3. These steel strips were removed after the initial setting (4 h) of concrete to simulate cracks at the dangerous sections of the pipe. Cracks made with this method are of the same shape, which can ensure that the deflecting conditions of all specimens are consistent. The specimens were demolded after 24 h and then cured for 7 days before the TEBTs. Noting that the surface preparation is usually applied in constructions to enhance the bonding quality—whereas the manual chipping process on the surface is difficult to control the roughness of the chipped surface—after the treatment, the roughness factor of the surface (i.e., JCR or fractal dimension of the surface) should also be considered as variable. In our study, we were more concerned about the impact of the lining material and the dimension of the liner on the rehabilitating results, so we ignored the surface treatment to avoid uncertainties; the interface was untreated in both tests of CM and ER to keep the same surface condition.

Subsequent TEBT showed that the ultimate load of the undamaged RCP specimen (T0-1) reached 53 kN/m, which exceeds the highest criteria suggested in the Chinese standard GB/11836 [32] of 41 kN/m for 300 mm RCPs. This means the RCP produced in the laboratory is qualified for practical application, and the production process is guaranteed.

**Figure 4:**
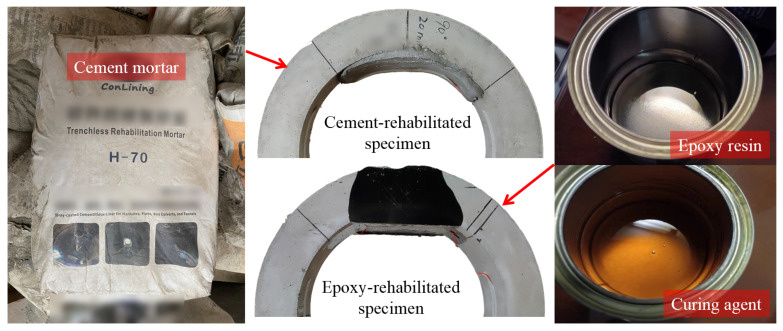
Cement mortar and epoxy resin used for pipe rehabilitation.

The CM and the ER materials used for the partial rehabilitation are shown in Figure 4. The common trenchless rehabilitation mortar and the normal ER material with the curing agent were selected as the rehabilitation material. In the rehabilitation, the ER and the curing agent were 1:1 mixed, and it could be cured in 2 h; the mixture was dyed black since a laser range finder (LRF) was used to control the lining thickness and the transparency of the resin greatly disturbed the result. For the RCP rehabilitated with ER shown in Figure 4, the lateral surface of the pipe crown was also black. Since the resin is highly fluid before it is cured, a minor part of it will leak into the narrow gap between the pipe and the molding plate and form a very thin layer (within 0.5 mm) at the lateral surface; its impact on the load bearing capacity of the pipe is limited.

#### 2.2.2. Test Instruments

**Figure 5:**
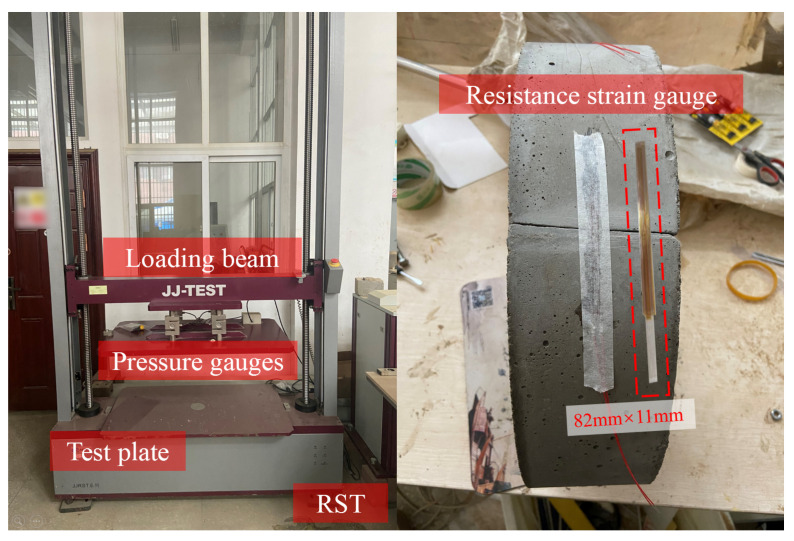
RST and resistance strain gauge used in TEBTs.

After the specimens were well rehabilitated and cured, they were then tested with a ring stiffness tester (RST), as shown in Figure 5. The RST can test pipes with a diameter of 110–2000 mm and a load of up to 50 kN, it is also equipped with a displacement acquisition module and four pressure gauges to measure the real time load and the displacement during the TEBTs; the accuracy of both the displacement and the load measurement was 0.5%.

In addition, resistance strain gauges were used to measure the strains at dangerous sections (the crown, invert, and springlines) of the RCPs during TEBTs. As shown in Figure 5, the sensitive grid length of the strain gauge was 82 mm, which was far larger than the crack width, and it could well reflect the strain changes at the test points.

### 2.3. Test Procedures

**Figure 6:**
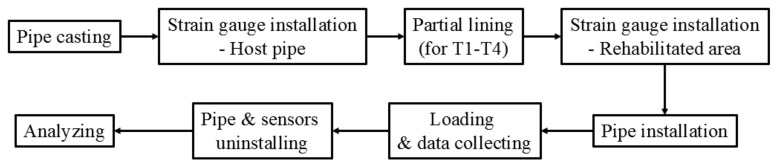
Main procedures of TEBTs.

The procedures of the TEBTs are shown in Figure 6. The key procedures included the strain gauge installation, the RCP installation, loading, and data acquisition, which will be detailed in this section.

#### 2.3.1. Strain Gauge Installation

**Figure 7:**
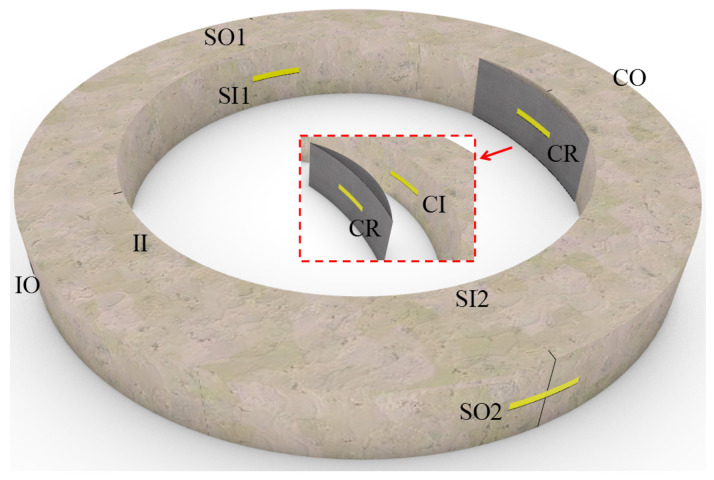
Positions and codes of strain gauges.

Before and after the partial lining, strain gauges at different positions were installed to the RCPs. Before the rehabilitation, strain gauges both inside and outside of the crown, invert, and springlines were installed, the codes of which are specified in Figure 7. After the partial liner was cured, the last strain gauge (i.e., CR in Figure 7) was installed at the surface of the liner.

#### 2.3.2. Pipe Installation

**Figure 8:**
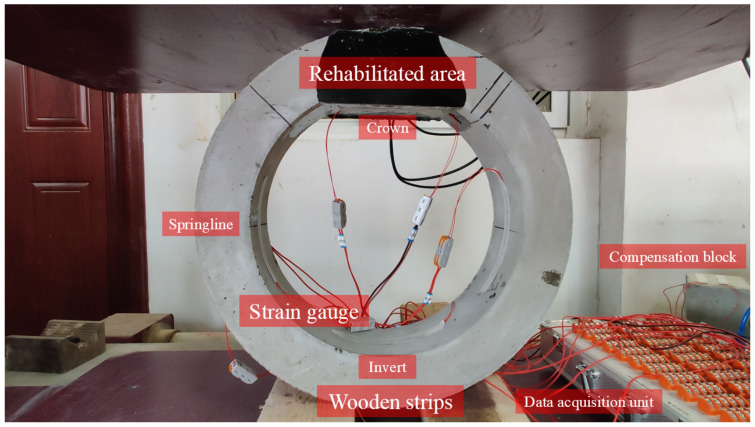
Layout of pipe installation in TEBTs.

The installation of the RCP follows ASTM C497-19 [33]: the pipe was supported by two parallel wooden strips with a distance of 100 mm, as shown in Figure 8, then the strain gauges and the compensation block were connected to the data acquisition unit, and the strain gauges were calibrated before loading.

#### 2.3.3. Loading

**Figure 9:**
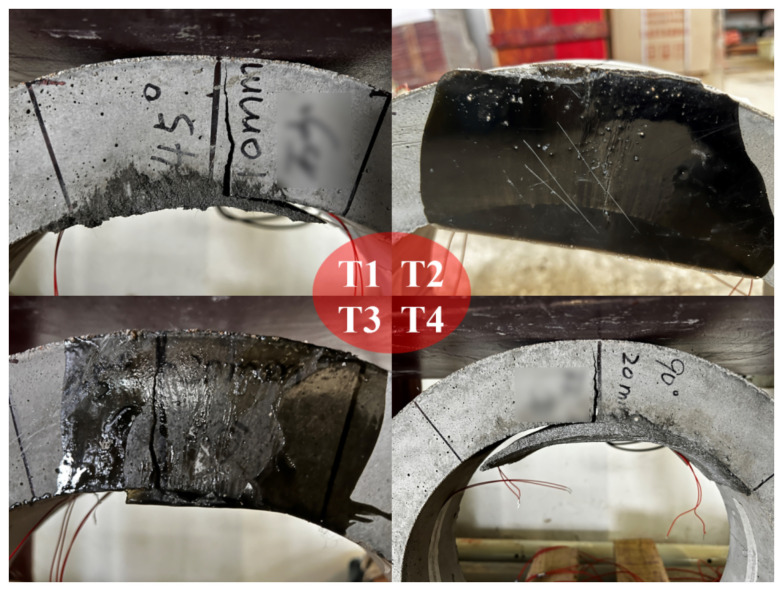
Liner–pipe interfaces after TEBTs of T1–T4.

All specimens were loaded with a constant rate of 5 mm/min, the critical time points of T1–T4 TEBTs are listed in Table 2. The practical loading duration was approximately 2 min in all test groups, which suggests that an ultimate displacement of 10 mm is common even if the materials and the dimensions of the partial liners are different; the failure mode of the defective RCP was not been changed by the rehabilitation. It can be learnt that the ER lining can delay the time that cracking happens; especially for test T2, the onset of cracks was effectively delayed. In addition, the bond between the ER liner and the RCP is also much stronger than the CM liner, as shown in Figure 9 and Table 2. For RCPs rehabilitated with CM, the liners separate from the pipes as the crack propagates (T1 and T4), whereas the ER liners hardly get separated from the pipes during TEBTs.

## 3. Results and Analysis

### 3.1. Load-Displacement Curves

**Figure 10:**
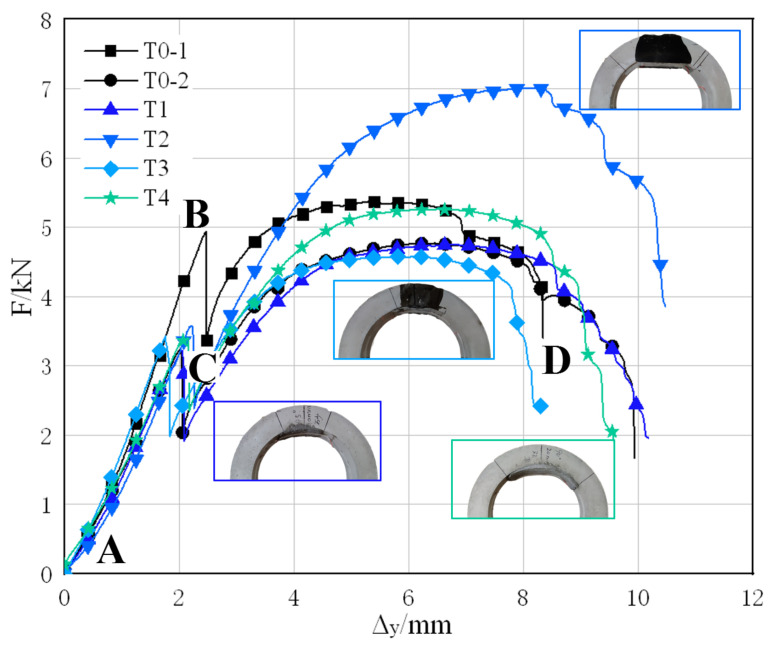
Load-displacement curves of TEBTs. (AB. Initial deflecting; BC. Pipe collapse; CD. Plastic deflecting).

The load-displacement curves of the 6 TEBTs are shown in Figure 10, all curves show a similar trend of elastic and inelastic deflecting. Take the curve of T0-1 (black curve with square label in Figure 10) as an example, the initial phase AB, BC, and CD represent different stages. In the initial deflecting phase (phase AB in Figure 10), the load and the vertical displacement are linearly related, although it appeared like an elastic deflection, there are still minor cracks generating and propagating in the structure, and as the vertical displacements reach approximately 2 mm, the pipe collapses due to the crack propagation (phase BC in Figure 10), and the load-displacement curves drop steeply. Then, the plastic deforming governs the deflection (phase CD in Figure 10): where the tensile stress in the reinforcement cage is the major resistance to the F-load, the compressed part at the dangerous sections of RCP form plastic hinges, and the cracks develop rapidly. As a result, when the tensile stress in the reinforcement at dangerous sections reaches the yield strength, the pipe collapses.

In Figure 10, two black curves represent the undamaged RCP (T0-1) and the defective RCP without rehabilitation (T0-2). It can be learnt that the failure load of the intact RCP is 5.37 kN; and, for the RCP with four cracks, the failure load is 4.77 kN, 11.17% less than the intact pipe. The colored curves represent TEBTs T1–T4, respectively, among which the T2 RCP has the highest failure load of 7.01 kN, which is 46.96% higher than that of the defective pipe and even 30.54% higher than the failure load of an intact pipe. The failure load of T4 RCP also reaches 5.26 kN, basically restoring the load-carrying capacity of an intact pipe. However, the RCPs of T1 and T3 only have a failure load of 4.74 kN and 4.58 kN, respectively, which is the same level of defective RCPs, and it cannot show the effect of rehabilitation.

Analysis of the load-displacement curves above shows that the lining thickness and the lining range have a decisive effect on the rehabilitation result; even if the liner does not form into an intact ring, it can still improve the residual bearing capacity of the defective RCPs. RCPs of rehabilitation condition 2 (20 mm lining thickness and 90° lining angle) have restored the load-carrying capacity of an intact pipe, whereas the load-carrying capacity of RCPs with rehabilitation condition 1 (10 mm lining thickness and 45° lining angle) has little improvement. On the other hand, the lining material also greatly influences the result, the ER material has a higher tensile strength and a better interface bonding strength than CM, so it can better perform at the tensile zone of the pipe crown. The failure load of RCP rehabilitated with ER under condition 2 increased by 46.96%, and the failure load of CM rehabilitated RCP under the same condition increased by 10.27%.

### 3.2. Load-Strain Curves

**Figure 11:**
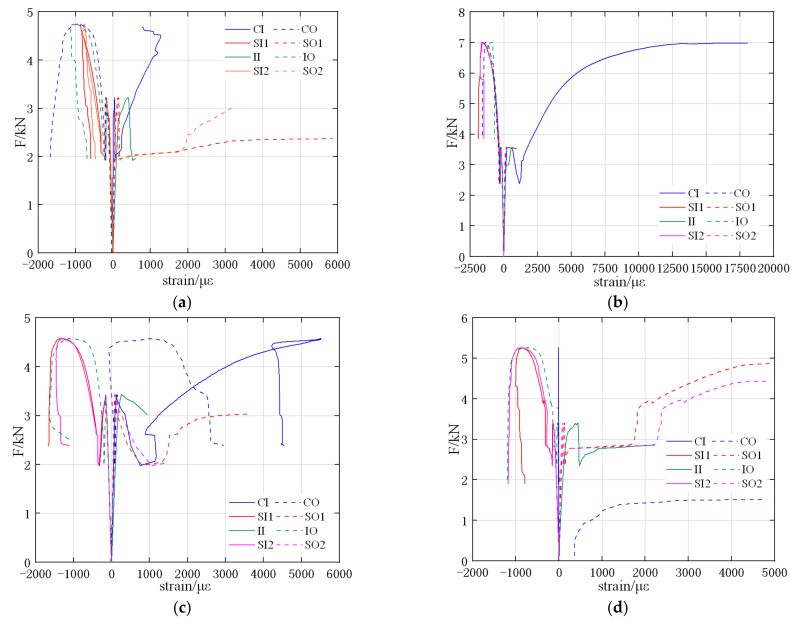
Load-strain curves of TEBTs. (**a**) T1; (**b**) T2; (**c**) T3; (**d**) T4.

The load–strain curves of T1–T4 RCPs are shown in Figure 11, respectively, where the negative values of strain indicate a compressive strain and the positive values indicate a tensile strain. At the tensioned positions (i.e., internal surfaces of crown and invert and external surfaces of springlines), nearly all strain gauges are ruptured due to the crack propagation; for these positions, the load–strain curves only include the part before that where the strain gauges are broken.

All load–strain curves of T1–T4 show a similar trend of elastic and inelastic phases: in the elastic phase, the loads and the strains are linearly related, and the strains are mostly in 300 με, which is negligible. In the plastic phase, the strains begin to increase rapidly, especially the tensile strains. This is also caused by the crack propagation and plastic collapse of the RCPs. It can be found in Figure 11b that the strain at the inner crown of RCP is much larger than in Figure 11a,c,d, which means this strain gauge is not torn apart by the crack development; the bonding quality at the inner crown of T2 specimens is much better than the other three specimens.

### 3.3. The Deforming Compatibility of the Partial Liner and the Pipe

**Figure 12:**
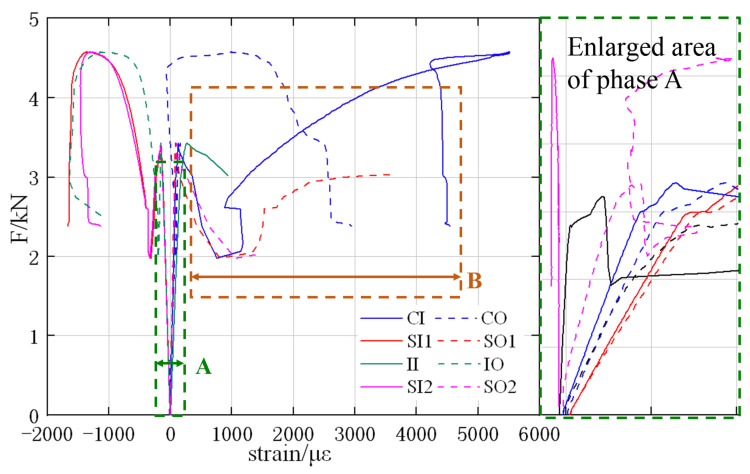
Comparison between the CI and the CR strains.

In this section, the circumferential strains at the internal surface of pipe crown (denoted as CI) and central surface of partial liner (denoted as CR) are compared and analyzed, the result of which can reflect the deforming compatibility of the partial liner and the pipe. The load–strain curves of strain gauge CI and CR in RCP T1–T4 are shown in Figure 12.

The load–strain curves include the elastic phase (phase A in Figure 12) and the inelastic phase (phase B in Figure 12); phase A has been enlarged so that the curves are better distinguished. It can be learnt from phase A that the red curves (solid line and dashed line) are quite close to each other and so are the blue curves, indicating that the liners and pipes of T2 and T3 (both rehabilitated with ER) have a better deforming compatibility than the other groups; the ER liner–pipe interface shows a stronger bond strength than the CM liner, which is also consistent with the experiment results.

In the inelastic phase (phase B), it is found that the red curves (T2) still have a good consistency, while the blue curves (T3) start deviating from each other, which means that a larger lining range can also effectively improve the deforming compatibility, even if the pipe is plastically deformed.

### 3.4. The Flexural Rigidity Increasing of the Partial Lined Section

Flexural rigidity (EI) is a major index to evaluate the bending performance of the section, which can also reflect the load-carrying capacity of RCPs. In this section, the EI of defective and rehabilitated crown sections of RCPs are calculated and compared.

By using conservation of energy, the relationship between moments and *F*-load can be established as [34]:

(1) $$F=\frac{M_{cr}+M_{in}+M_{sp1}+M_{sp2}}{R}$$

where F is the *F*-load of TEBT, N; $M_{cr/in/sp1/sp2}$ are moments at the crown, invert, and springlines, respectively, N·mm; and R is the average radius of RCP, mm. The relationship between the moment and the EI of each section can be established as follows [35]:

(2) M=κEI

(3) $$\kappa =\left(\varepsilon_{i}-\varepsilon_{o}\right)/t$$

where κ is the curvature of the section; $\varepsilon_{i}$ and $\varepsilon_{o}$ are the internal and external strains, respectively; t is the wall thickness of RCP, mm; and EI is the flexural rigidity of the section, N·mm^2^.

With the test data of the T0-2 group, the original EI of the defective section (denoted as $EI_{o}$) can be calculated with Equations (1)–(3), consequently, for T1–T4 groups, the EIs of rehabilitated crown sections (denoted as $EI_{r}$) can be calculated with:

(4) $$EI_{r}=\left[FR-EI_{o}\left(\kappa_{in}+\kappa_{sp1}+\kappa_{sp2}\right)\right]/\kappa_{cr}$$

where $\kappa_{cr/in/sp1/sp2}$ are the curvatures of sections at crown, invert, and springlines of the RCP, respectively. The calculated $EI_{o}$ and $EI_{r}$ are shown in Table 3.

It can be found that the partial lining method can effectively improve the EI of RCPs. Even for groups T1 and T3, whose residual bearing capacities are not significantly improved, the EIs of their rehabilitated crown sections increased to 2.64 and 1.6 times that of the defective section, respectively. For T2 and T4 with larger lining thickness and range, the EIs of their rehabilitated crown sections increased to 9.29 and 6.02 times that of the defective section, respectively.

## 4. Conclusions

According to the test and the analysis results, the following conclusions can be drawn:

(1) Structural problems such as cracking and corroding are common in municipal tunnels, such as cable tunnels, in major cities in central and eastern China. However, the pipelines, shelves, and other auxiliary facilities in the tunnel will shelter the lateral walls, making it difficult to rehabilitate an intact ring with traditional trenchless methods—partial lining is a feasible, potential method for rehabilitating these structures;
(2) The results of TEBTs for partial lining rehabilitated RCPs indicate that applying the appropriate material and dimensions of a partial liner can effectively improve the load-carrying capacity of RCPs. The failure load of RCP specimens rehabilitated with partial ER lining increased by 46.96% compared to the defected RCP, which is 30.54% higher than the failure load of an intact pipe. The failure load of RCPs rehabilitated with CM partial lining also increased by 10.27%, basically restored to the level of an intact pipe;
(3) As the lining material, epoxy resin has a better deforming compatibility with the original pipe, while the cement mortar liner will gradually separate from the pipe as the is crack propagating, thus, using the fiber reinforced cement mortar (FRCM) is a better solution to improve the tensile strength and the bonding quality of the liner. Besides, a larger lining thickness and lining range can also strengthen the interface performance between the liner and the pipe, making them work in better coordination;
(4) The flexural rigidities of all rehabilitated sections reach 1.6–9.3 times that of the defective section, even for those groups whose load-carrying capacity is not significantly improved, their flexural rigidities also obviously increase after the partial lining rehabilitation;
(5) According to the comprehensive results of TEBTs and analysis, the partial lining method is feasible for structural rehabilitation in municipal tunnels. It is also a convenient, environmentally friendly, and cost-efficient method, and it has the value of being popularized. At present, the research on this method is still blank; how to further improve the load-carrying capacity of the liner-pipe composite structure and design the dimensional parameters of the partial liner is also a good research interest with great potential.

## Figures and Tables

**Table 1:** Designing and coding of TEBTs.

Code	Defects	Lining Material	Lining Angle	Lining Thickness	
T0-1	No defects	N/A	N/A	N/A	Control groups
T0-2	10 mm cracks	N/A	N/A	N/A	
T1	10 mm cracks	CM	45°	10 mm	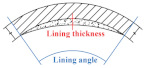
T2	10 mm cracks	ER	90°	20 mm	
T3	10 mm cracks	ER	45°	10 mm	
T4	10 mm cracks	CM	90°	20 mm	

**Table 2:** Critical time points in T1–T4 TEBTs.

	Onset of Crack	Liner Breakdown	Pipe Collapse
T1	19 s	22 s	122 s
T2	27 s	Not separated	125 s
T3	20 s	97 s (slightly)	100 s
T4	17 s	30 s	115 s

**Table 3:** Flexural rigidities of defective and partially rehabilitated sections.

Test	$\kappa_{cr}/{\mathrm{mm}}^{-1}$	$\kappa_{in}/{\mathrm{mm}}^{-1}$	$\kappa_{sp1}/{\mathrm{mm}}^{-1}$	$\kappa_{sp2}/{\mathrm{mm}}^{-1}$	$EI_{o}/(N\cdot {\mathrm{mm}}^{2})$	$EI_{r}/(N\cdot {\mathrm{mm}}^{2})$	$EI_{r}/EI_{o}$
T0-2	7.61 × 10^−5^	6.79 × 10^−5^	13.11 × 10^−5^	6.82 × 10^−5^	5.07 × 10^9^	-	-
T1	5.02 × 10^−5^	2.90 × 10^−5^	6.86 × 10^−5^	11.17 × 10^−5^	5.07 × 10^9^	1.3 × 10^10^	2.64
T2	4.20 × 10^−5^	3.20 × 10^−5^	3.74 × 10^−5^	4.52 × 10^−5^	5.07 × 10^9^	4.7 × 10^10^	9.29
T3	9.30 × 10^−5^	4.30 × 10^−5^	8.89 × 10^−5^	4.86 × 10^−5^	5.07 × 10^9^	8.1 × 10^9^	1.60
T4	2.01 × 10^−5^	5.70 × 10^−5^	9.97 × 10^−5^	10.13 × 10^−5^	5.07 × 10^9^	3.1 × 10^10^	6.02

## Data Availability

Some or all data, models, or code that support the findings of this study are available from the corresponding author upon reasonable request.
